# A Wearable System for Remote Wrist Skin Temperature Monitoring to Assess Skin Thermal Response to Extreme Cold: A Case Study

**DOI:** 10.3390/s26103235

**Published:** 2026-05-20

**Authors:** Jakub Janowicz, Grzegorz Wiczyński

**Affiliations:** Institute of Electrical Engineering and Electronics, Poznań University of Technology, Piotrowo 3a Street, 60-965 Poznań, Poland; grzegorz.wiczynski@put.poznan.pl

**Keywords:** skin temperature, correlation, varying environmental conditions, thermal stress

## Abstract

Skin temperature measurement is a complex issue. Skin tissue is one of the main thermoregulatory organs and takes major responsibility for heat exchange in the organism. Accurate skin temperature measurement may contribute to better estimation of deep core temperature, which is why enhancing possibilities of skin temperature measurement is considered substantial. However, the real value of the skin temperature can be influenced by many biological and non-biological factors. Some of the external factors such as extensive wind or extreme ambient temperature may significantly influence the raw value of the skin temperature regardless of the choice of the measuring point. Despite that, abnormal thermoregulatory behaviour can occur due to internal body stresses and reactions. Whilst internal influence is even more difficult to track than external factors, it is crucial to monitor and identify the thermal stresses in a correct way. The paper proposes a wrist temperature measurement system. The system consists of a sensory part placed in a housing adapted to the shape of the wrist. The sensory component enables contact measurement of wrist skin temperature under the assumed experimental conditions. The housing is designed to provide stable positioning of the sensory component relative to the wrist while simultaneously isolating it from external conditions. The paper presents the results of a case study concerning human thermoregulation, quantifying the thermal response of the hand under low-temperature exposure in temperature chamber and during the subsequent rewarming phase after removal. During the experiment, temperature measurements of both hands were recorded. One of the co-authors participated in this case experiment. The temperature measurement results were compared between the hand subjected to thermal stress and the hand not exposed to low temperatures. Differences in the participant response to repeated thermal stress are demonstrated. The results highlight the complexity of the human body’s thermoregulation process in extremely cold environments.

## 1. Introduction

Temperature plays a significant role in the human body. A significant change in deep core temperature can suggest various diseases. In a healthy body, the deep tissues maintain a constant temperature in the range of (37 ± 0.5) °C regardless of the ambient temperature [1]. This is due to the thermoregulatory mechanisms developed in the process of evolution [1,2]. The major part of the body heat exchange takes place in three parallel processes: radiation, convection, and evaporation [3]. Since the most popular non-invasive ways of estimating deep core temperature include measuring skin surface temperature, it is important to explore skin thermal behaviour and routines [4,5,6,7,8].

The skin takes partial responsibility for all the aforementioned processes, while being indispensable in heat exchange by evaporation [3]. The contribution of radiation heat dissipation to overall heat transfer varies in the range of (40–60)% and directly depends on the difference between the surface temperature of the skin and the temperature of the surrounding medium [3,7,9]. About (15–20)% of the heat is transferred based on the convection phenomenon due to the flow of air molecules around the body [3,7]. Evaporation from the skin and lungs is the last of the major heat exchange mechanisms [3]. It is responsible for about (15–20)% of all heat transfer [1]. The phase transition energy of the fluid must be delivered from the body simultaneously lowering its energy [1,2,3].

The substantial role of the skin in these processes may, and should, cause its temperature to vary considerably over time [7,10]. The complex fundamentals underlying the mechanism make it necessary to take into account multiple biological, physiological and non-physiological factors in order to explain the origin of the variability [3,10]. The skin temperature ($T_{S}$) may also vary a lot based on the choice of the measurement point. In the distal (placed relatively far from the heart) regions of the body (e.g., hands, feet) $T_{S}$ is in general lower than in the proximal regions (e.g., chest, forehead), especially when taking into account vasomotion in peripheral cutaneous vascular plexuses [1,2].

So far, there has been a lot of research done, concentrated on $T_{S}$ measurement in proximal areas for clinical applications. This parameter can effectively estimate deep core temperature in stable, favorable environmental conditions, with the method inaccuracy even below 0.1 °C [4,8,11]. However, it may be problematic in terms of application. That is why it is crucial to develop measuring systems with more appealing application possibilities such as wrist temperature sensors [12].

Nowadays, the knowledge on distal $T_{S}$ is wide [13]. There are many publications proving that correlation of distal $T_{S}$ with other quantities can enhance the accuracy of deep core temperature accessible estimation [1,11,12,13,14,15]. The relationship does not have to be neither deterministic nor direct; however, it gives a valuable insight especially in multimodal systems. The technological advancement of $T_{S}$ measuring devices has recently moved forward, presenting a possibility of a contact, non-battery, non-chip measurement with an electronic skin-thin device [16]. Despite the great innovation these solutions brought to biomedical engineering, the accuracy of e-skin temperature measurement remains unverified due to its direct exposure to surroundings, especially in varying environmental conditions [17]. In order to fully understand the variability of $T_{S}$ and be able to interpret it correctly, the behavioural principles of $T_{S}$ under stresses needs to be verified and unambiguously proved. This can be done only by supporting a sensing device which goal is a correct, conditions insensitive $T_{S}$ measurement. Some of the recent publications regarding $T_{S}$ response to thermal stresses are described below.

In [18] the authors observe the body’s response to wrist thermal stress. The volunteers put their hands into cold, 15 °C water while sitting in room temperature for 1 min. Then the thermal response and recovery time were monitored with IR camera. The limitations of this study arise from the measurement method which is not vastly accurate. However, it is a good point of reference for evaluating heating recovery time.

In [19] very similar research was done but after taking the wrist out of the water, the hand was irradiated with an optical radiation. The external heat source caused the wrist to heat up more quickly; however, the results strictly depended on the research subject and were mostly divergent.

The authors of [20] presented an evaluation of human perceptions of heat and cold during the exposure to different thermal stimuli. They studied hand palm thermal response; however, they measured it only in the beginning and in the end of the study. The main focus of this work was to establish a conclusion about temperature sensation, not the measurement itself. However, the research shows that evaluation of human body behaviours and reaction to stresses is an actual issue.

The problem of hand $T_{S}$ measurement and the interpretation of its variability is an important and current issue. This research paper proposes measuring system for stable, accurate and wireless wrist $T_{S}$ measurement, capable of reliable identification of wrist $T_{S}$ response to cold stress. Subsequently, it carries out an experiment testing skin thermal response to harsh environmental conditions. In order to meet the legal requirements of ethical committee regarding research including human volunteers in Poland, the author was the only subject of the study [21]. The document has been divided into three sections. Section 2 of the document introduces the complete design of the system and experimental protocol. Section 3 presents complete results of the experiment, the discussion and the comparison with current state-of-the art in the field of skin temperature measurements.

## 2. Methodology

**Figure 1:**
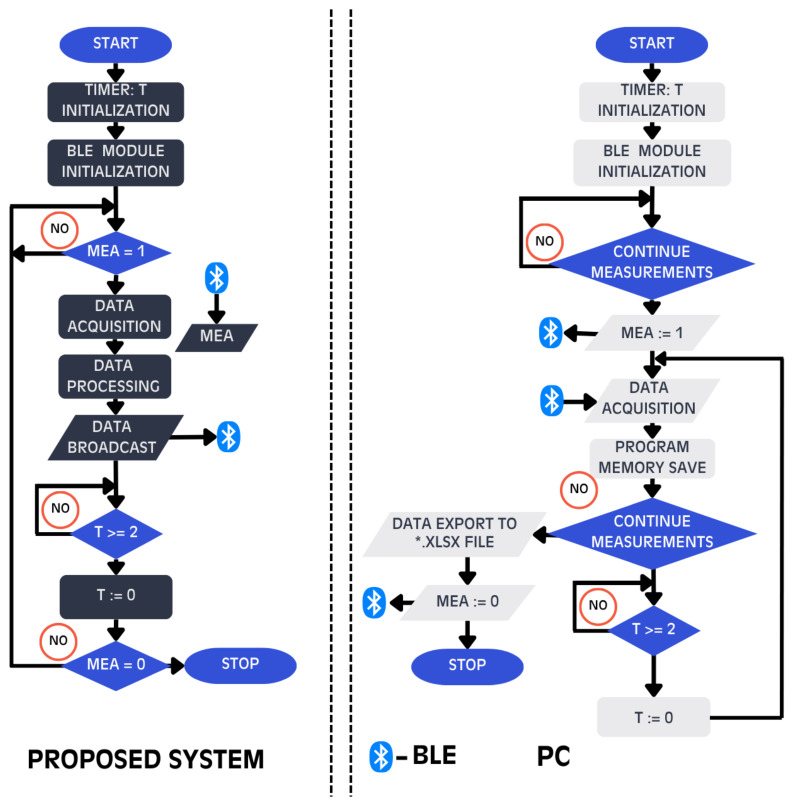
Operation algorithm of the measurement system and PC application. There are two auxiliary variables implemented (T as timer value in seconds and MEA as a boolean variable controlling measuring state).

This case study aims to verify the usage of a proposed system to identify thermal response variability of both wrists in varying thermal conditions. Author himself was the only object of the study. The research covered an observation of both wrists’ $T_{S}$ during two environmental cases. In one case the author was sitting in a room with a stable ambient temperature ($T_{A}$) at room temperature level. The second case considered assumption that one wrist (left) was exposed to low temperatures (from −10 °C to 2 °C) in a thermostatic chamber, whereas the torso and the other wrist (right) remained in a room temperature. The design of the system and the conduct of an experiment are described below. The operation algorithm of the system is described in Figure 1.

### 2.1. Electronical Design

**Figure 2:**
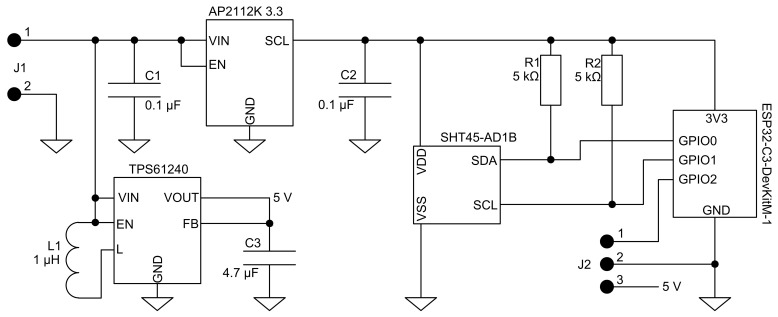
Scheme of the electronic part of the proposed system.

Figure 2 shows the complete electronics schematic of the circuit. The data acquisition was done by a capacitive humidity and temperature sensor prod. by Sensirion of type SHT45-AD1B. It is a digital sensor communicating via the $I^{2}C$ bus with a manufacturer-declared accuracy of 0.1 °C in the temperature range (5–70) °C with the resolution of 0.01 °C. However, owing to the executed calibration, the system can be effectively used outside of the manufacturer-declared temperature range. The system uses BLE (Bluetooth Low Energy) protocol for starting and stopping acquisition as well as data transfer between the system and PC. It was used due to its low energy consumption comparing with WiFi in ESP32 family microcontrollers. Due to the assumed minimization of the number of components, it was decided not to use additional external memory. An external PC application was designed, to provide live data storage and presentation. The control was designed based on ESP32-C3-Mini microcontroller using a ESP32-DevkitM-1 development board. The system was powered by a 3.7 V Li-Po battery prod. by Cellevia Batteries with a capacity of 1850 mAh.

The system performs temperature acquisition every 2 s, simultaneously updating the current temperature on the PC display.

### 2.2. Mechanical Design

**Figure 3:**
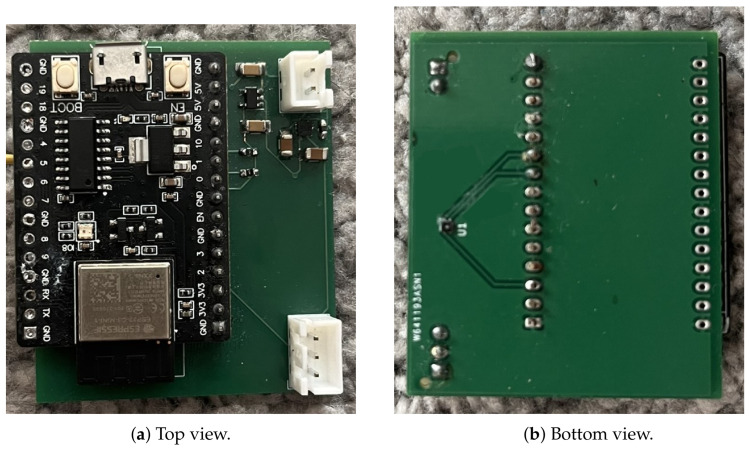
View of the measuring system PCB.

**Figure 4:**
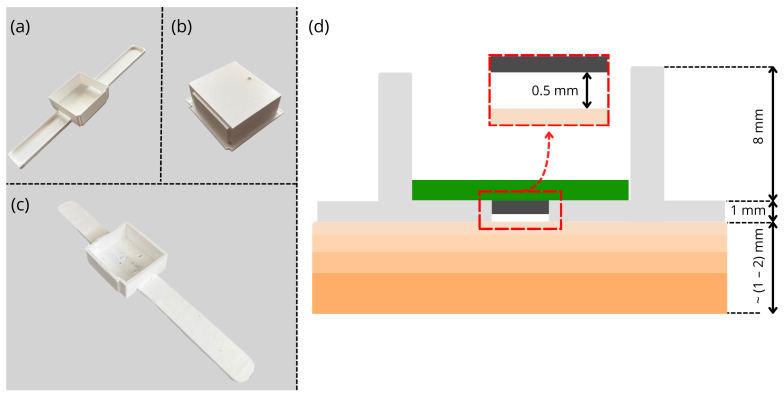
View of the: (**a**) silicone casting mold, (**b**) filling for side-wall casting, (**c**) physical implementation of the housing. (**d**) Cross-section of the housing and skin layers.

**Figure 5:**
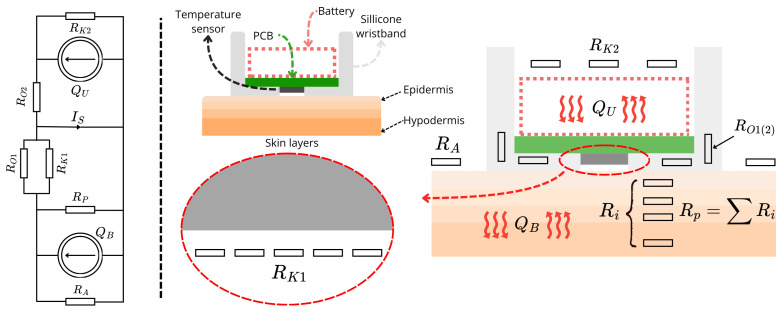
Conceptual heat flow diagram with cross-section view of the system; where: $Q_{B}$—source representing human body heat generation, $Q_{U}$—source representing proposed system heat generation, $R_{K1/2}$—thermal resistance of the air for the heat generated by the body/electronic system, $R_{O1/2}$—thermal resistance of the silicon band for the heat generated by the body/electronic system, $R_{A}$—thermal resistance of the air for the heat generated by the body, $I_{S}$—heat transferred to the sensor.

The complete system consisted of the electronic circuit (Figure 3) and the wristband housing (Figure 4). The PCB was designed to fit in the wrist case and was printed on FR4 board. The housing was designed to allow highest possible accuracy and stability of the system, simultaneously remaining easy in application and comfortable to wear. Additionally, the goal was to ensure thermal isolation from the air. Conforming to these limitations, flexible, all-purpose silicone was used as a housing material. However, regarding low thermal conductivity of the silicone (approx. (0.2–0.6) $\frac{W}{\mathrm{mK}}$ [22]) it was necessary to improve thermal conduction between the surface of the wrist skin and the housing-sensor structure. To enhance the heat transfer, two copper wires were embedded into the silicon band and the bottom part of the housing. The wires were formed into a flat wick with a cross-sectional area of 1 mm^2^ and placed along the entire length of the band. The case was designed to fit the electronic circuit and the battery. Since both the electronic circuit and the battery generate heat during operation, there was a strong likelihood of the sensory setup self-heating. To prevent this and allow best possible heat exchange between the processing and battery system part and the air, the housing did not contain upper lid. To ensure stability and accuracy, the housing had a sensor slot on the bottom allowing the sensor to be at a tiny distance from the wrist skin surface. The assumed heat flow diagram of the system is presented in Figure 5. The assumed diagram is a conceptual model of the extensive heat flow. It was based on mathematically validated heat flow models of other skin-related devices [13,23,24].

### 2.3. Calibration

Prior to the experiment both systems were calibrated in order to ensure that they have convergent indications. A comparative method was used for calibration, by encapsulating both systems and a laboratory reference measurement standard in a custom modified thermostatic chamber dedicated for thermometer calibration, which is a property of an ILAC MRA signatory accredited laboratory with a CMC (Calibration and Measurement Capability) of 0.06 °C [25]. The results of the calibration are listed in the Table 1 as the measurement errors of the both systems against the measurement standard indication in a given calibration setpoint. The results are provided along with the standard deviation of the mean *SD* ($SD_{T_{L}}$ and $SD_{T_{R}}$ for left and right wrist system respectively) and the expanded uncertainty *U* (k = 2) established using the method used for thermometer calibration in an ILAC MRA signatory laboratory. The given uncertainty value corresponds to all the established errors. The calibration was executed for 30 min under repeatable conditions with sampling interval of 2 min. The temperature reading of left (cold) wrist system is labeled as $T_{L}$ whereas the temperature reading of right (warm) wrist system is labeled as $T_{R}$.

Since the designed circuit is not intended for use for fixed point measurements, three point calibration was executed covering the entire useful temperature range. Calibration data was then used to establish 2nd order polynomials for measurement error interpolation. The data presented below is already corrected for errors according to the established formulas for left wrist ($T_{WL}$) and right wrist ($T_{WR}$) measuring systems.

(1) $$T_{WL}=-0.0003\cdot {T_{L}}^{2}+0.0086\cdot T_{L}-0.2421$$

(2) $$T_{WR}=-0.0003\cdot {T_{R}}^{2}+0.0078\cdot T_{R}-0.2313$$

### 2.4. Robustness Against Environmental Disturbances

In order to verify the system’s overall dynamic performance it tested against environmental disturbances. The idea behind this experiment was to verify the thermal response of the system placed on a constant temperature cylindrical object with dimensions similar to the human wrist during thermal steady state and environmental disturbances.

For this purpose, a section of brass pipe was selected with the diameter of 70 mm and wall thickness of 2 mm. It was equipped with additional calibrated surface temperature sensor. The pipe was placed inside the thermostatic chamber, with a temperature setting of 20 °C, while the system remained outside until reaching the thermal steady state of the pipe and itself. Afterwards, the system was mounted on the pipe and remained still until reaching the thermal equilibrium—the state in which the temperature reading of the system did not vary more than 0.1 °C for more than one minute.

**Figure 6:**
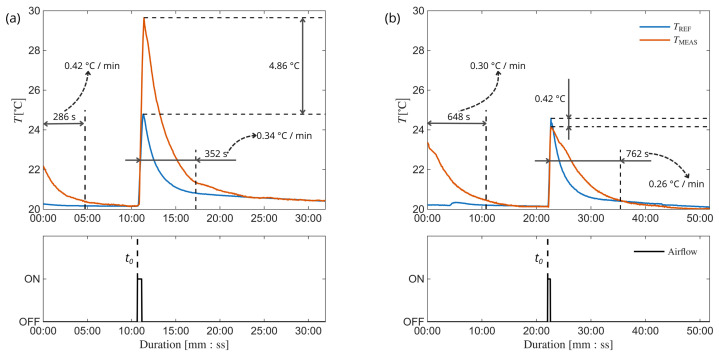
Thermal variability of the pipe and the designed system during external heat flow disturbance trial: without (**a**) and with (**b**) the housing.

Then, the additional heat and airflow disturbance was introduced to the system. The hairdryer was used to enforce the additional airflow around the pipe. The enforced airflow speed and temperature were monitored and equaled $\left(18.0\pm 0.3\right)\;m/s$ and $\left(31.03\pm 7.05\right)\;{}^{\circ}C$ respectively. To prevent uncontrolled heat exchange between the inner chamber and the environment, the chamber doorway was modified and the 3 cm thick polystyrene foam with a forearm wide hole in the middle was mounted to cover the entire entrance. The disturbance was enforced for around 30 s and the response of the pipe and the system was observed until reaching the thermal equilibrium afterwards. The same procedure was executed for the PCB without the designed housing and the PCB placed inside the designed housing. The experiment allowed for the recognition of response and recovery times and identification of the gain in the resistance to external disturbance. The entire course of the test is presented in the Figure 6 where $T_{REF}$ is the pipe temperature and $T_{MEAS}$ is the temperature measured by the tested system.

The entire protocol analysis can be clearly divided into two separate sections: before introducing the additional heat flow and after. First section analysis gives the information about the 90% rise time. Although the raw rise time value is much different between (a) and (b) cases, starting system temperatures were different at the beginning of the protocol. The slope is more informative in these circumstances and it can be seen that compared to the raw PCB, the introduced system lost around 25% of dynamics in terms of the response time. On the other hand, the results provided by the system with the housing were significantly closer to the pipe temperature after the disturbance occurred. Moreover, the Maximum Absolute Temperature Difference (MATD) between $T_{REF}$ and $T_{MEAS}$ was notably lower. As the final indicator of disturbance resistance Mean Absolute Error of Reading (MAE) was calculated for the second part of the study according to (3). Finally, at the expense of the dynamic response, the system’s MATD decreased over 10 times with MAE improved by over 55% comparing to the raw PCB circuit. The key test results were summarized in Table 2.

(3) $$MAE=\frac{\sum_{t=t_{0}}^{t_{END}}\left|T_{REF}\left(t\right)-T_{MEAS}\left(t\right)\right|}{\frac{1}{2}\left(t_{END}-t_{0}\right)}$$

### 2.5. Experimental Protocol

To verify the thermal behaviour two types of measurement series were done, both including two measuring systems (each mounted on one wrist). Firstly, the subject remained still in the room temperature, sitting on the chair. After 10 min of stabilization period the measurements began. This protocol was used two times to verify both wrists’ unilateral temperature variability and compare systems’ performance in stable, favorable environmental conditions. These series are called the test series. Subsequently, the main protocol was executed by conducting seven measuring series of $T_{WL}$ and $T_{WR}$ enclosing left wrist in a thermostatic chamber. The chamber used in the study incorporated the doorway modification described in Section 2.4, allowing the hand to be inserted without keeping the chamber opened. The experiment began with a 10 min stabilization period of $T_{WL}$ and $T_{WR}$ in room temperature prior to the beginning of measurements. During that time the temperature of −10 °C was already maintained inside the chamber. Given the similar environmental conditions during the trials, the initial external conditions can be considered comparable across the trials. Due to thermal losses through the polystyrene the temperature inside the chamber built up to about (1–2) °C during every measurement series. The inner chamber temperature variability was monitored with the external calibrated temperature sensor placed within 5 cm distance from the wrist. Although the variability was considerable, the conditions were both repeatable and reproducible, and therefore do not influence the conclusions.

The break criterion was the discomfort threshold of the author. Although it is not a standardized, repeatable criterion, this protocol allows the comparison of adaptation to cold exposure among different physiological well-being series. After the cooling period, the initial part of thermal recovery process was recorded, and then the acquisition was stopped.

## 3. Results and Discussion

### 3.1. Experimental Results

In the paper, some key parameters were monitored or calculated in order to examine the thermal behaviours of the sole subject’s body. The indicators taken into consideration are listed in Table 3 along with the qualitative relevance of the individual factors to the study. The relevance was assessed based on physiological features represented by the following parameters. Based on the aim of the study the highest priority was given to the features describing any kind of impact or correlation of $T_{WL}$ to $T_{WR}$, especially that $T_{WR}$ variability trend has never been examined in similar conditions before. Low relevance was assigned to the factors that do not describe the relation between $T_{WL}$ and $T_{WR}$ but focus on only one of the parameters instead.

Environmental conditions, covering ambient temperature ($T_{A}$) and ambient humidity ($RH_{A}$) were monitored during the study. The room was a property of a calibration laboratory, $T_{A}$ did not change by more than 0.5 °C and $RH_{A}$ did not change by more than 5% during an hour. Additionally, the well-being state of the subject was noted before the beginning of the study. The physiological data is collected in order to enhance possibilities of identifying the outlier behaviours. Physiological parameters measurement and correlation is not the subject of the study. All the additional data are listed in Table 4.

**Figure 7:**
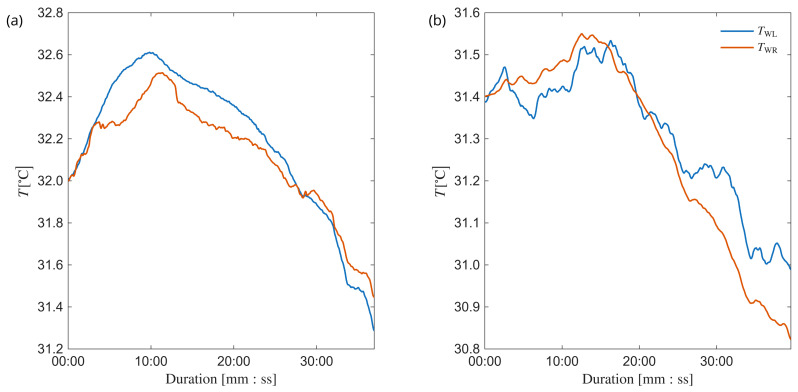
Temperature variability during the test series T1 (**a**) and T2 (**b**).

During both test series the object sat still in a room temperature. The left hand remained completely still, lying on the table during the measuring period whereas the right hand conducted some basic activities such as checking the phone or surfing on the Internet. In the second series, both hands were lying on the table without performing any additional activities. Both test series lasted about 35–40 min. The resulting temperature variability is shown in Figure 7.

**Figure 8:**
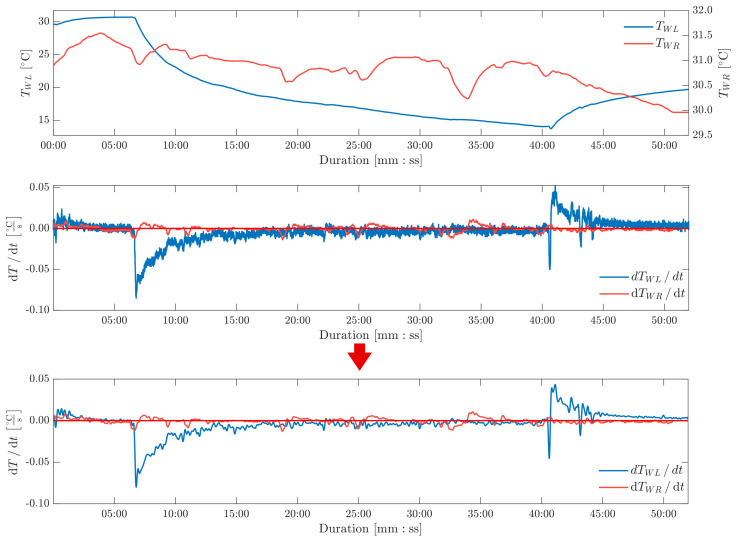
$T_{WL}$, $T_{WR}$ and their derivatives variability during the exemplary time series (No. 7) before DWT filtration (upper plot) and after (lower plot).

During proper study, the left hand was put into the thermostatic chamber. The measuring series differed in time due to the varying condition of the organism and pain threshold. The exemplary variation in $T_{WL}$ and $T_{WR}$ is shown in Figure 8 along with their first derivatives. In order to minimize measurement noise in the derivative plot, a moving average filter was used on the original signal, averaging four consecutive data points. Additionally, to smooth out the derivative of both signals, the high frequency noise was reduced by using maximal overlap discrete wavelet transform. Decomposition was done using symlet8 wavelet, decomposing it into five frequency components. The data presented regards only the residual, low frequency part. As Figure 8 depicts, the preprocessing does not change the signal overall trends and shape but significantly improves the readability and decreases noise.

The key evaluation factors listed in Table 3 were assessed separately for every measurement series.

Differences in the $T_{WR}$ readings were calculated by picking the highest and the lowest value of $T_{WR}$ measured in a time interval from 10 s before putting the hand into the chamber until 1 min after taking the hand out of the chamber. $T_{WL}$ variability was assessed from the during cooling period. The comparison between both test series and the measuring series is presented in the Table 5 along with the cooling time and $T_{WL}$ as well as $T_{WR}$ variability. The maximum variability has been highlighted in green. The test series in the table were sorted by the cooling time in the ascending order. To have a better insight into the relationship between $T_{WL}$ and $T_{WR}$ pearson correlation coefficient was calculated and presented in a Table 6.

**Figure 9:**
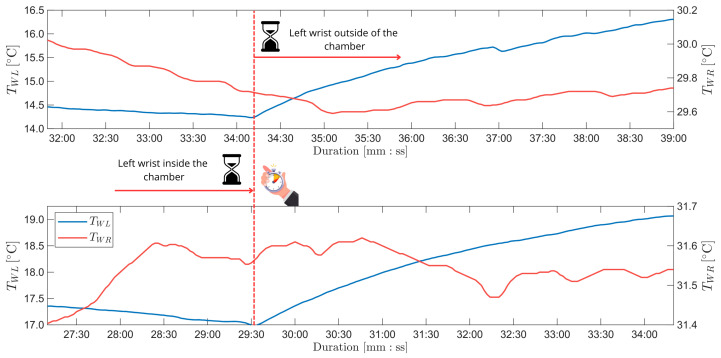
The time course of the end of the cooling phase and the beginning of the heating phase of the series number: 4 (top chart) and 6 (bottom chart).

After removing the left hand from the thermostatic chamber the temperature was still monitored. The cold wrist temperature monotonically rised during this period. The other wrist’s thermal behaviour was varing from series to series. In Figure 9 two divergent behaviours are shown. $T_{WR}$ in the heating part was neither monotonically rising nor monotonically decreasing. The general variation trend was not unified through all the series either. The monotonicity of $T_{WR}$ in the heating phase for each study is listed in Table 7 (I stands for increasing, D stands for decreasing).

The $T_{WL}$ first derivative settling time was established by calculating the time window from the beginning of cooling/heating to the time moment in which the derivative was oscillating around the established value. In some of the series the heating period has not established an oscillatory constant $T_{WL}$ derivative value during the measuring period. The settling times for $T_{WL}$ derivative are presented in Table 8.

### 3.2. Comparison with Current State of the Art

The system proposed in the paper can be used to conduct an effective, wireless skin temperature measurement. It is operating in a wide range of external conditions that allows monitoring $T_{S}$ in extraordinary situations.

Comparing to current state-of-the art the device does not constitute a technical novelty in terms of mechanical or electronical advancement. The novel wrist sensors combine $T_{S}$ measurement with GSR (Galvanic Skin Response) and heat flow measurement [26,27,28,29]. What is more, novel systems allow accurate core body temperature estimation in stable environmental conditions or in clinical trials with a low estimation error at the level of 0.2 °C [26,28]. However, systems based on heat flux sensors often require stable environmental conditions and focus on deep core temperature estimation from $T_{S}$ measurement instead of $T_{S}$ measurement itself what makes them vulnerable to harsh environmental surroundings [30,31].

On the other hand, the current research interests lead to development of multimodal wrist or finger measuring systems, capable of simultaneous measurement of many biological signals, e.g., PPG, BVP and skin temperature [32,33]. The implementation of such systems allows multimodal analysis that can bring holistic information about the body condition, but it usually does not provide a credible $T_{S}$ value.

The main advantage of the proposed system over the currently available solutions is the complete focus on accurate $T_{S}$ value measurement, allowing for a reliable measurement in difficult external conditions—making the solution suitable for thermal stress response recognition. It might be of interest for the future studies to replace the sensory element with a thermoelectric sensor to improve the dynamic response of the system [34].

Referring to commercially available solutions there are a few medical grade, wrist-worn devices that handle skin temperature measurement [35,36]. However, most of them are not supposed to be used in a temperature range below 0 °C [37,38,39]. It is therefore substantial to further validate the prototype against the medical-grade devices in the future, with an ambient temperature maintained above 0 °C.

### 3.3. Discussion

The proposed measuring system has been proven to provide valid skin temperature readings in difficult environmental conditions. The systematic error components were diminished by fitting the results to the calibration curves. The custom housing design significantly improved the system performance by reducing vulnerability to external heat-flow disturbances, which reduced the random error components. Moreover, the calibration standard uncertainty was at the level of 0.03 °C, calibration errors were convergent over the entire temperature range and the thermal response of the system to heat-flow disturbance was almost linear to the thermal response of the measured object. All that allows for assessing the system credibility as high.

The system was used to analyze the distal thermal response of the body to thermal stress of the left wrist. This is a particularly important need because it gives a pilot insight into the human thermoregulatory response to long exposure to extreme cold.

The first, apparent conclusion is that without external thermal stresses, the thermal behaviour of distal areas (wrists) is convergent, on the contrary to the stress situation. In Table 6 only test series coefficients allow to classify the correlation between both wrists’ $T_{S}$ as very high. In the other cases it differs from the moderate, to high correlation, excluding the no. 3 series (negligible correlation).

Secondly, combining the information provided in Table 4, Table 5 and Table 6 it can be inferred that the well-being of the organism significantly influences the distal thermoregulatory process. In the series no. 3 (which corresponds to the worst well-being among all) the measured and calculated metrics diverge from the others. During this series occur: the biggest temperature difference between both wrists, the lowest correlation between $T_{WR}$ and $T_{WL}$ and the lowest obtained $T_{WL}$ value (subjectively the most mild pain perception).

Moreover, the behaviour of $T_{WR}$ after removing the left wrist from the chamber is ambiguous. As presented in Figure 9, the $T_{WR}$ reaction to $T_{WL}$ condition changes differed amongst measuring series. The overall behaviour regarding full observation time (listed in Table 7) is non-deterministic and does not correlate to any of the measured quantities. Hence, the reason of this phenomenon may be rather internal.

Lastly it should be noticed, that the cooling time did not specifically determine the ΔT value neither in case of $T_{WR}$ nor $T_{WL}$. Table 5 was sorted in the cooling time ascending order but the values of ΔT are not aligned in the ascending order. It suggests the contribution of body internal factors, especially in case of $T_{WL}$, which was acquired from the wrist, kept in stable conditions in the thermostatic chamber. Similar conclusion can be drawn in relation to settling time (Table 8). However, the settling time estimations (especially the ones regarding heating process) should be established considering a larger time interval, so its relevance is low (Table 3).

### 3.4. Limitations

The authors identify two major and one minor limitation valuable to address in future work. The major limitation is the case character of the study. The limited number of participants prevents from drawing high-fidelity, general conclusions about human thermal regulation. On the other hand, the single-subject study focused on intra-personal analysis that could be conducted due to reproducible environmental conditions. Despite this limitation, the originality of the results highlights a research gap in thermal studies involving extreme cold exposure.

The second identified limitation is the lack of numerical analysis regarding the proposed heat flow model. Neither the simulations nor the numerical calculations have been provided to validate the model or estimate the proposed model parameters. The model is just an assumption and requires future validation. Lastly, the study lacks quantitative validation of the influence of copper conductors incorporated into the housing. Although the positive influence of the entire housing on the robustness of the system has been proven, the influence of the copper conductors themselves requires attention in the future in order to further optimize the system’s performance.

## 4. Conclusions

The paper presents a developed system for continuous monitoring of wrist skin temperature. This system was used to conduct a case study on human thermoregulation, determining the thermal response of a volunteer. The study involved placing one of the volunteer’s hands in a thermal chamber (−10 °C) and then removing it from the chamber (room temperature). The other hand remained at room temperature. Both wrists’ skin temperature was monitored during the experiment. The temperature measurements of the hand exposed to thermal stress were compared with the hand not exposed to low temperatures. Differences in the volunteer’s response to repeated thermal stress were observed. The measurement results confirm the complexity of the human body’s thermoregulatory process in an extremely cold environment. This necessitates continued pilot studies combining the fusion of distal skin temperature measurements and other biomedical signals involving a larger group of volunteers.

## Figures and Tables

**Table 1:** Calibration results.

Measurement Error [°C]	Calibration Point [°C]		
	−10	0	35
$T_{L}$	0.36	0.24	0.26
$T_{R}$	0.34	0.23	0.26
$SD_{T_{L}}$	0.004	0.002	0.002
$SD_{T_{R}}$	0.004	0.001	0.002
*U*	0.06		

**Table 2:** Environmental disturbance experiment key results.

Parameter	Results	
	Without Housing	With Housing
Slope $\left[\frac{{}^{\circ}C}{\min}\right]$	0.42	0.30
$MATD\;\left[{}^{\circ}C\right]$	4.86	0.42
$MAE\;\left[{}^{\circ}C\right]$	0.74	0.33

**Table 3:** Research evaluation factors and their relevance to the study.

No	Factor	Relevance
1	Difference between $T_{WR}$ in the beginning and the end of the study	HIGHEST
2	Direct correlation of $T_{WL}$ and $T_{WR}$	HIGH
3	$T_{WR}$ changes character (i.e., monotonicity, reaction time) after the left wrist removal from the thermostatic chamber	MEDIUM
4	$T_{WL}$ first derivative settling time during cooling and heating period	LOW

**Table 4:** Environmental conditions and psychophysical state of the research subject before each study.

No	$T_{A}$ [°C]	${\mathrm{RH}}_{A}\;\left[\%\right]$	Psychophysical State
1	21.5	57.4	No signs of disease, relative pressure feeling normal, not feeling tired, 25 min after drinking a coffee
2	22.1	52.8	Observable signs of disease (mild runny nose), not enough sleep at night, relative pressure feeling normal, elevated heart rate (reading from a smartwatch prod. by Garmin of type Venu 3)
3	21.7	56.2	Maintaining running nose and blockage of the airways, relative pressure feeling normal, heart rate normal
4	22.0	49.8	Good well-being, relative pressure feeling normal, heart rate normal, minor wound on the left wrist
5	22.4	54.3	
6	20.9	51.9	
7	19.4	51.2	

**Table 5:** Analysed variability of $T_{WL}$/$T_{WR}$ before and after the cooling period (series 1–7) and $T_{WR}$ during both test series (T1, T2).

**No**	$T_{\mathrm{WR}}$ **[°C]**			$T_{\mathrm{WL}}$ **[°C]**			**Cooling Time [s]**
	**Max**	**Min**	ΔT	**Before**	**After**	ΔT	
T1	32.51	31.45	1.06				
T2	31.55	30.82	0.73				
1	32.38	31.55	0.83	33.58	16.55	17.03	1610
6	32.56	31.16	1.40	32.46	17.00	15.46	1720
4	31.46	29.69	1.77	31.36	14.48	16.88	1840
2	31.67	30.35	1.32	31.59	13.73	17.86	1900
5	32.90	31.27	1.63	33.50	14.91	18.59	1940
7	31.32	30.23	1.09	30.70	13.72	16.98	2030
3	30.77	29.16	1.61	32.26	13.56	18.70	2240

**Table 6:** Pearson correlation coefficients between $T_{WL}$ and $T_{WR}$ time series.

T1	T2	1	2	3	4	5	6	7
0.987	0.979	0.775	0.591	0.043	0.498	0.575	0.605	0.803

**Table 7:** Evolution trend of $T_{WR}$ during the heating part.

1	2	3	4	5	6	7
I	D	I	I	D	D	D

**Table 8:** $T_{WL}$ derivative heating and cooling settling times.

Phase	Settling Time [s]						
	1	2	3	4	5	6	7
Heating	-	-	-	380	600	530	560
Cooling	370	380	410	300	400	610	600

## Data Availability

Data will be provided upon request.

## Abbreviations

The following abbreviations are frequently used in this manuscript:

$T_{A}$	ambient temperature
$RH_{A}$	ambient humidity
$T_{S}$	skin temperature
$T_{L}$	raw left-wrist skin temperature reading
$T_{WL}$	corrected left-wrist skin temperature reading
$T_{R}$	raw right-wrist skin temperature reading
$T_{WR}$	corrected right-wrist skin temperature reading
MATD	maximum absolute temperature difference
MAE	mean absolute error
